# Knowledge, attitude and practice related to chemical hazards and personal protective equipment among particleboard workers in Ethiopia: a cross-sectional study

**DOI:** 10.1186/s12889-019-6807-0

**Published:** 2019-04-27

**Authors:** Akeza Awealom Asgedom, Magne Bråtveit, Bente Elisabeth Moen

**Affiliations:** 1 Centre for International Health, Department of Global Public Health and Primary Care, Faculty of Medicine, University of Bergen, Bergen, Norway; 2 Ethiopian Institute of Water Resources, Addis Ababa University, Addis Ababa, Ethiopia; 3 Department of Global Public Health and Primary Care, Faculty of Medicine, University of Bergen, Bergen, Norway

**Keywords:** Attitude, Chemical hazard, Knowledge, Particleboard factory, Personal protective equipment, Permanent worker, Practice, Temporary worker

## Abstract

**Background:**

Work in the wood industry is often associated with exposure to wood dust and formaldehyde. The aims of this study were to describe the Knowledge, Attitude and Practice (KAP) concerning chemical health hazards among particleboard workers and to compare the KAP among temporary and permanent workers.

**Methods:**

A cross-sectional study design was used to collect data by structured questionnaires in two particleboard factories in Ethiopia. A total of 159 workers and 13 management personnel participated in this study. Both closed-ended and open-ended questions were included in the interviews. Chi-square tests, *T* tests and correlation analyses were used for categorical and continuous data. Total knowledge score (range 0–8) was calculated as the sum score of 8 items weighing one point each. Multiple linear regression was applied to estimate the impact of employment status on total knowledge score adjusted for level of education. Content analysis was applied to analyse collected data from open-ended questions.

**Results:**

The mean age of the respondents was 28 (*SD* = 6) years and on average they had 3.7 [3] years of service. The permanent workers were older than the temporary workers (29 vs 26 years, *p* = 0.001), and a considerably high fraction of the permanent workers had vocational education (90%) compared to the temporary workers (11%). Permanent workers had higher proportion of response on knowledge of 10 of 12 topics regarding chemical hazards and attitudes on 6 of 11 of these topics than temporary workers. Permanent workers had higher knowledge scores (3.7) compared to temporary workers (1.3) (*p <* 0.001), also after adjusting for education (*p* = 0.011). Permanent workers were provided with personal protective equipment (PPE) while temporary workers were not. The qualitative data helps to understand the workers and administrative personnel attitude and thinking regarding chemical hazards and PPE.

**Conclusions:**

The findings revealed that permanent workers have higher proportion of positive response on knowledge and attitude towards chemical health hazards than temporary workers. However, practice in use of PPE depended on access to PPE. Few temporary workers were provided with PPE.

**Electronic supplementary material:**

The online version of this article (10.1186/s12889-019-6807-0) contains supplementary material, which is available to authorized users.

## Background

Particleboard is a wood product which is increasingly produced and used in Ethiopia. It is manufactured from lignocellulosic materials, primarily in the form of discrete particles, combined with urea formaldehyde resin and bonded together under heat and pressure. Particleboard is used, for instance in production of office tables, shelves and interior wall partitioning [1, 2]. The manufacturing sector, comprising wood, metal, food, textile, leather and construction industries, accounts for 6.9% of the national work force in Ethiopia [3].

Work in the wood industry is associated with exposure to wood dust [4–11], and in the particleboard industry the workers might also be exposed to formaldehyde from glue resin [12–14]. Exposure to wood dust may cause acute irritation of the skin, eyes and airways [15, 16] and may also be associated with chronic respiratory symptoms [16–18]. Formaldehyde may also cause respiratory problems [14, 19]. Wood dust and formaldehyde are classified as carcinogenic (Group 1) by International Agency for Research on Cancer [20, 21].

The hierarchy of occupational hazard control from the most effective to the least effective can be described as: Elimination, substitution, engineering control, administrative control and PPE [22]. To reduce exposure to wood dust, the most effective control measures may not be present, or not work sufficiently. As a result, in many workplaces PPE is recommended as an immediate control measure, as the expense of providing PPE is relatively low and can quite easily be provided. The cost of face mask, coverall, glove, and other PPEs is covered by the employer. Workers in the wood industry are recommended to wear appropriate face masks and eye protection in areas with high dust and formaldehyde exposure. Coveralls and industrial gloves are needed to protect the skin [23, 24].

It is important for the workers to be informed about the health hazards and why control measures are necessary. Otherwise, workers do not always wear PPE, even in high risk situations at work. However, information alone might not be sufficient to change the attitude and practice of workers. A model called “Knowledge, Attitude and Practice” (KAP) has been developed to describe and understand these challenges better. The KAP model consists of a triad of interactive factors [25] and can help us to understand why the workers do not adhere to specific advice or rules by evaluating their behavioural determinants [26].

A study done in the United States revealed that use of PPE was negatively affected by lack of comfort and fitness, young age and lack of safety training [27]. Studies have shown that the use of PPE varies from 10 to 82% depending on accessibility, adequacy, affordability, fitness to the user and its discomfort [28–32]. A study done in Nigeria indicates that workers’ adherence to use of PPE was low because of shortage, inconvenience and the perception of PPE as unnecessary. Safety training played a significant role in increasing knowledge about PPE and health problems in the wood industry [33].

KAP studies done among farm workers in Ethiopia showed that 85% of the workers do not receive training on chemical pesticides, only 10% of the workers were using full PPE and the attitude and practice of handling chemical pesticides were poor [34]. The knowledge level of the participants on safety issues was affected by gender, safety training and work regulations [35]. Furthermore, use of PPE was affected by safety training, education, work regulation and their knowledge of safety information [35, 36]. In the textile industry, employment status was a determinant for PPE use, since permanent workers apply safe practice to a greater extent than temporary workers [35].

There are several gaps in occupational safety and health in Ethiopia, such as lack of trained manpower, weak implementation of policy and regulation and limited research, all of which reduce the possibility of identifying, assessing and controlling hazards. This shows us that there is a long way to go to address occupational safety and health [37].

The knowledge among workers in the Ethiopian wood industry about exposure to dust and formaldehyde and their health effects has not been studied. More knowledge on KAP is needed for implementation of control measures in this type of industry. Another aspect is that in the particleboard factory, as well as in other industries in Ethiopia, there are both permanent and temporary workers. The number of temporary workers is in general increasing and several studies show that they are at higher risk of occupational injuries and diseases than permanent workers [38–42].

The aims of this study were to describe the KAP concerning chemical health hazards among particleboard workers, with focus on their use of PPE and to compare the KAP among temporary and permanent workers. It is hypothesized that temporary workers are less protected than permanent workers. Studying the KAP in this industry is important in planning preventive measures to reduce health problems related to chemical hazards.

## Methods

**Fig. 1:**
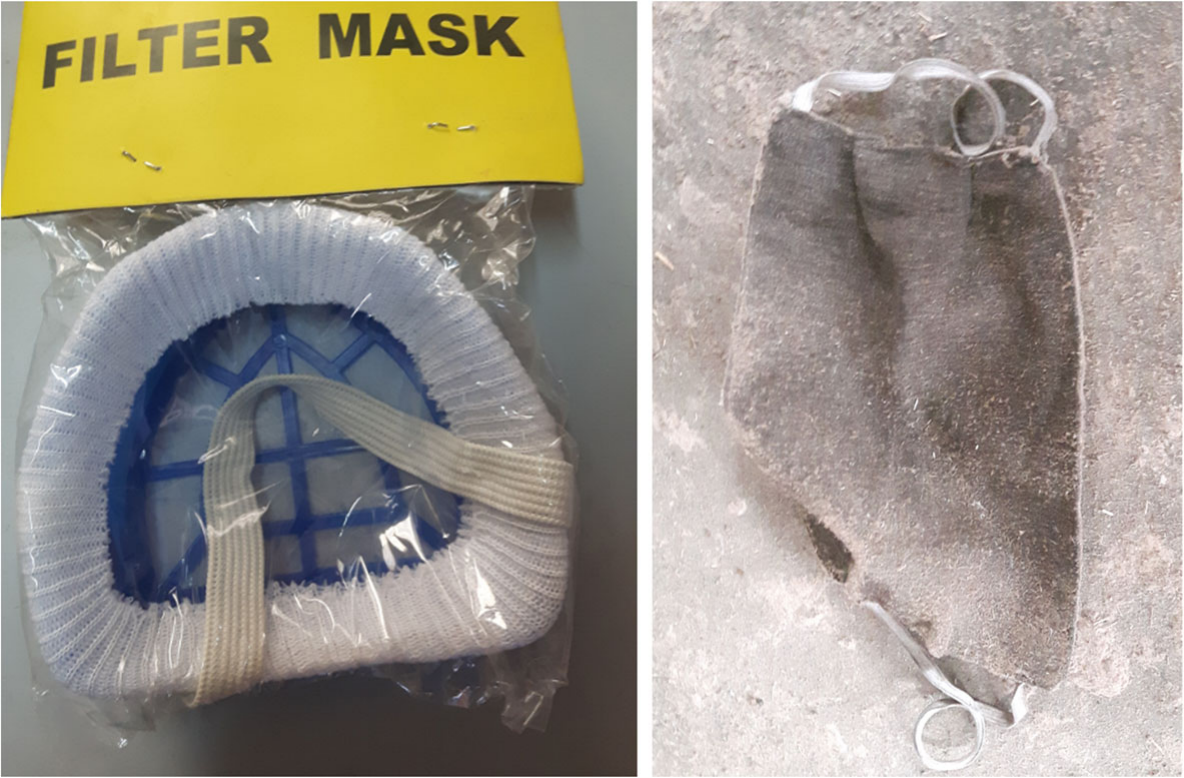
Face mask currently in use among particleboard workers in Ethiopia

A cross-sectional study design was used to collect structured questionnaire-based data from two of the largest particleboard factories in Ethiopia. The factory situated in northern Ethiopia has 663 workers and was established in 2005. The factory located in southern Ethiopia was established in 2002 and has 249 workers. The production lines in these factories are similar, comprising 10 sections: chipping, flaking, drier, boiler, blending, forming, pressing, trimming, sanding and sizing. In addition, there are workers with miscellaneous tasks who are working in all sections: cleaners and workers in the machine control room [1, 2], quality control and maintenance. The face mask currently used as personal protective equipment is shown in Fig. 1.

Production workers were the source of population for the study. The required number of participants was calculated with the purpose of describing use of PPE, using a single population proportion taking into account a 54% practice of using PPE obtained from a study done in Ethiopia among textile workers [35]. The output of the formula with 95% confidence interval, 5% level of significance and finite population correction gave 167 workers.

To plan the study, the factories and its leadership were visited. After obtaining permission to perform the study, we asked the management to provide the list of workers in each work shift (morning, evening and night). There were 8 working hours per shift.

Study participants were interviewed in a quiet and private place near their work by 10 trained bachelor environmental health professionals. After the interview the participants were also allowed to give their own comments about the working environment.

The questionnaire employed for data collection was developed by reviewing KAP questions from published articles in textile, petrochemical and other industries [26, 35, 43]. The questions were constructed in a way that addresses the hazards expected from the wood industries in the study. The proportion of questions were; 31 closed ended and 19 open-ended for workers and 12 closed ended and 12 open-ended for administrative personnel. Qualitative information was collected from worker and management staff using open-ended questions. The full data collection questionnaire is found as Additional file 1 for this article. The researcher also performed a workplace visit to observe the actual use of PPE and the type of PPE.

Information was collected from the workers in November and December 2016 using a structured questionnaire-based interview asking for sex (M/F), age (years), education (highest grade completed), profession, employment status (permanent/temporary), working section, number of service years, total working hours per day. In addition, the main body of the questionnaire contains knowledge, attitude and practice-related questions with no (N) or yes (Y) response options and some open-ended questions as indicated in as additional file 1. Completeness of the questionnaire and consistency was checked at the end of each day of the data collection.

The interview was based on qualitative and quantitative questions prepared in English and translated to Amharic by a translator, and then translated back from Amharic to English by another translator, to check the consistency. Pre-testing of the questionnaire was done on 5% of the sample population in one of the factories before the main study. Due to this test some questions were modified slightly before starting the actual data collection. Data were coded and entered in EpiData version 3.1.

A knowledge score was calculated as the sum score of 8 items weighing one point each. This score (0–8) consisted of knowledge of relevant chemical hazards at their workplace (2 items: dust and formaldehyde), relevant health effects from the chemical hazards (3 items: respiratory, eye and skin problems) and recommended personal protective equipment (3 items: coverall, face mask and gloves).

### Ethics

The study received ethical permission from regional committee for Medical and Health Research Ethics, Western Norway on June 2, 2016 with IRB ref.: IRB00006245 and from the Ethiopian Ministry of Science and Technology on October 7, 2016 with Ref. No. 3.10/148/2016. Written consent from the study participants and consent from factory management was assured before data collection.

### Statistics

Data was exported from EpiData version 3.1 to the statistical package SPSS, version 25 for analysis. Chi-square tests were used for comparing categorical variables. *T* tests were used to compare means of continuous variables. Correlation was used to analyse the association between knowledge score, age and service years. Multiple linear regression was used to analyse the association between employment status (permanent vs. temporary) and total knowledge score while adjusting for variables significantly associated with knowledge score in univariate analysis (*p* <  0.05). Content analysis was applied to analyse collected data from open-ended questions. The qualitative data provides supplementary information from administrative personnel on the general working environment, chemical hazards and PPE.

## **Result**s

### General characteristics of the study population

From 167 people invited, 159 (95%) workers (89 and 70 from the two factories) responded to the questionnaire. The remaining 5% of the respondents did not want to participate in the interview. In addition to the data collected from the production workers, qualitative information was collected from 13 management personnel (7 and 6 from the two factories).

There was no statistical difference between the employees from the two factories in terms of sex distribution (*p* = 0.3), age (*p* = 0.078), service years (*p* = 0.097), and consequently the data from the two factories were merged in the following analysis. However, educational status was significantly different between the two study sites (*p <* 0.001).

The arithmetic mean age of the respondents was 28 (*SD* = 6) years and the average service years of the respondents was 3.7 [3] years. Eight people had worked in another similar factory with service years ranging from 1 to 20. The majority of the respondents among both permanent and temporary workers were men (94% vs 87%). The permanent workers were older than the temporary workers (29 vs 26 years, *p* = 0.001), and among the permanent workers a considerably higher fraction had at least vocational education (90%) than among the temporary workers (11%) (Table 1).

**Table 1:** Demographic characteristics of permanent and temporary particleboards workers in Ethiopia

Variable		Total *n* (%)	Employment status (*n* = 159)	
			Permanent (*n* = 121) *n* (%)	Temporary (*n* = 38) *n* (%)
Sex	Male	147(92)	114(94)	33(87)
	Female	12(8)	7(6)	5(13)
Education	Grade 1–10	46(29)	12(10)	34(89)
	Vocational and above	113(71)	109(90)	4(11)
Service year	Mean (*SD*)	3.7(3.0)	4(3.0)	2.5(2.4)
Age	Mean (*SD*)	28(6.0)	29(6.0)	26(5.0)

### Knowledge about chemical hazards

**Fig. 2:**
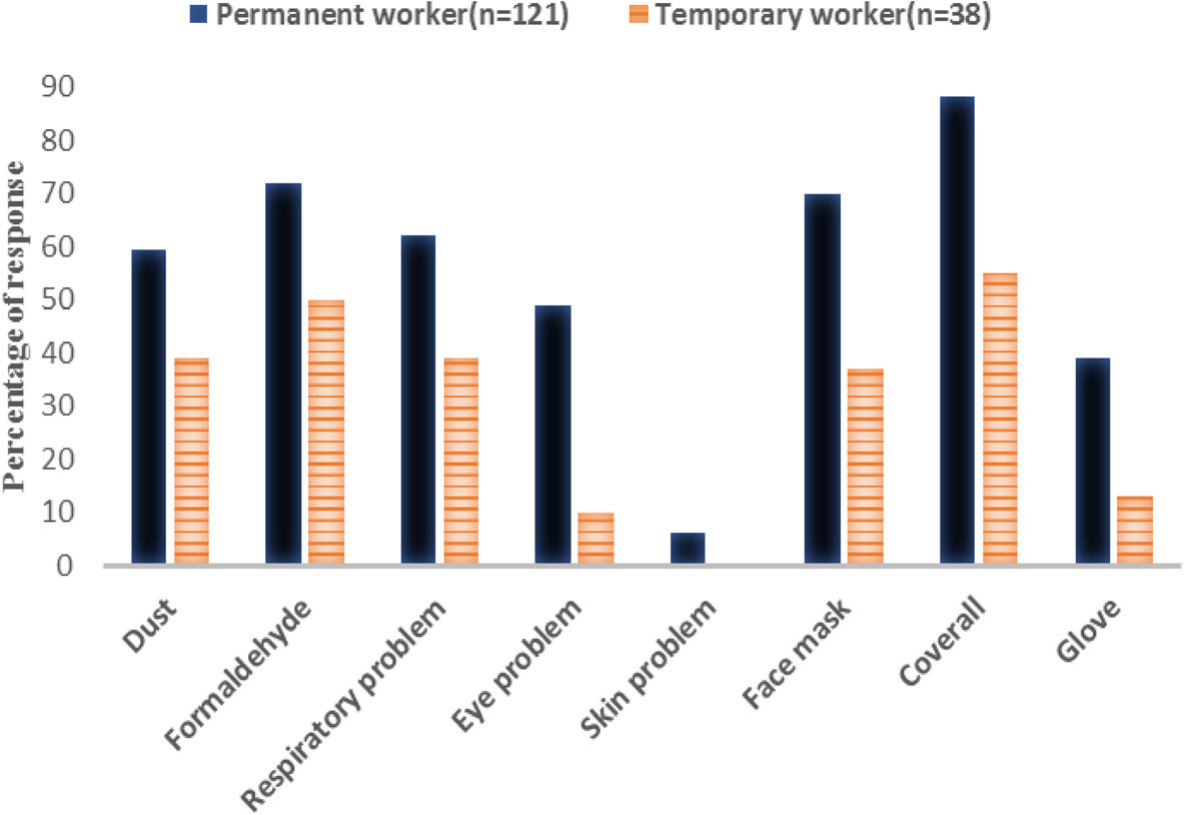
Type of chemical hazards, health problem and personal protective equipments stated by permanent and temporary particleboard workers in Ethiopia

**Fig. 3:**
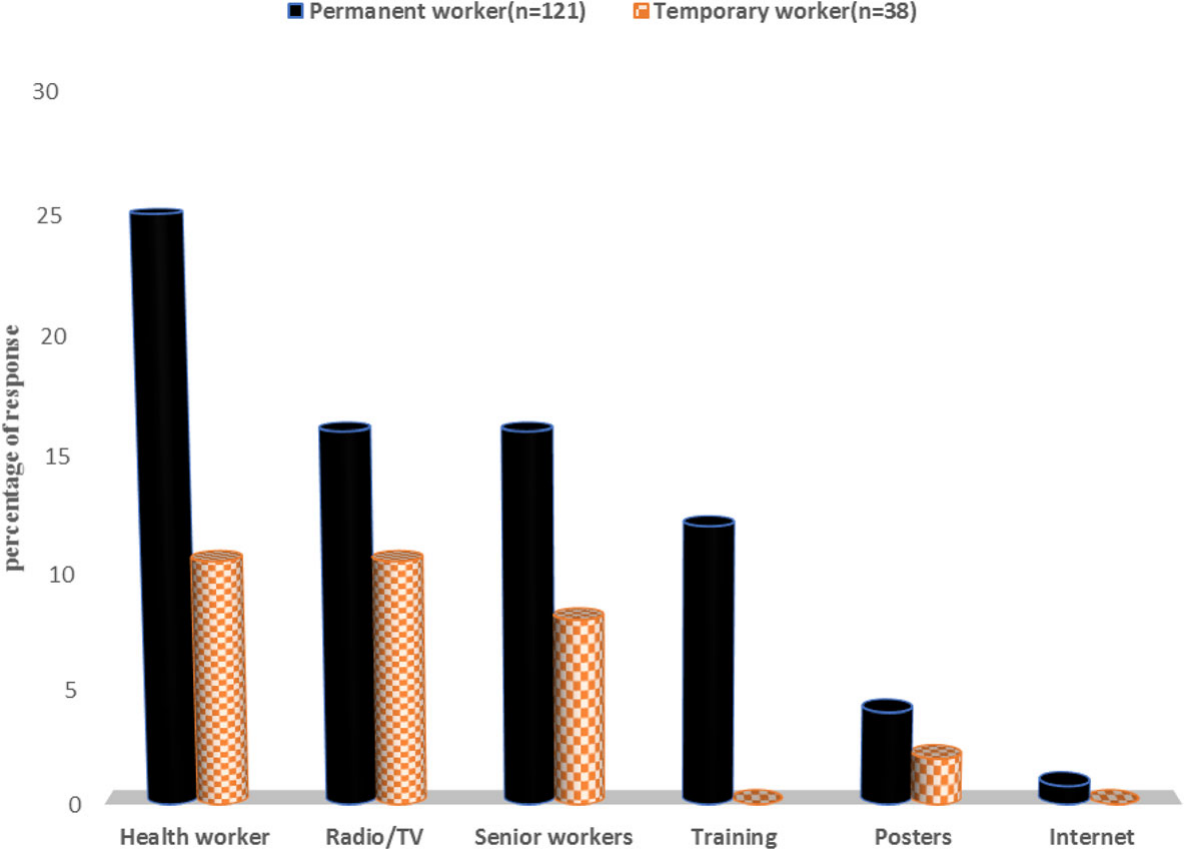
Sources of information about occupational health among permanent and temporary particleboard workers in Ethiopia

Permanent workers had significantly more knowledge than temporary workers about 10 of total 12 topics related to chemical hazards (Table 2). A high fraction of the permanent workers had knowledge of some chemical hazards (87%), health effects (80%) and relevant PPE (100%). Formaldehyde was the chemical factor mentioned by the highest fraction of both permanent and temporary workers (Fig. 2). Respiratory problems were mentioned more often than eye problems, while only a few workers mentioned skin problems. Coveralls, followed by face mask and gloves, were mentioned most often as relevant PPE. The primary sources of information about occupational health mentioned by the highest fraction of permanent workers were health workers, senior workers and radio/TV (Fig. 3). Only four of the temporary workers mentioned any sources of such information. Few permanent workers got information from the Internet.

**Table 2:** Knowledge about chemical hazards and protective measures among permanent and temporary particleboard workers in Ethiopia

Variable	Total *n* (%)	Employment status(*n* = 159)		*p* value
		Permanent (*n* = 121) *n* (%)	Temporary (*n* = 38) *n* (%)	
Know some chemical hazards	130(82)	105 (87)	25 (66)	0.007
Know some health effects	114(72)	97 (80)	17 (45)	*<* 0.001
Know some relevant types of PPE	144(91)	121(100)	23(61)	*<* 0.001
Know hazards other than chemicals	115(72)	93 (77)	22 (58)	0.038
Emergency exit is important	84(53)	81 (67)	3 (8)	*<* 0.001
Know material safety data sheet	35(22)	34 (28)	1 (3)	0.002
Know/understand sign and symbols of safety	75(47)	71 (59)	4 (11)	*<* 0.001
Know any safety rule in this workplace	89(56)	83 (69)	6 (16)	*<* 0.001
Know job rotation reduces exposure to chemical hazard	113(71)	97 (80)	16 (42)	*<* 0.001
Know break time during work reduces exposure to chemical hazard	140(88)	108 (89)	32 (84)	0.59
Have information on occupational health	63(40)	59(49)	4(11)	*<* 0.001
Know the factory has obligation to maintain workers’ health	143(90)	110 (91)	33 (87)	0.676

In univariate analyses employment status and education level were both significantly associated with the knowledge score while sex and age were not (Table 3). Mean knowledge score was 3.7 (*SD* = 2.4) among permanent and 1.2 (*SD* = 2.1) among temporary workers, respectively. There was no correlation between the knowledge score and service years (*r* = 0.015; *p* = 0.847) or between the knowledge score and age (*r* = 0.049; *p* = 0.452).

**Table 3:** Association between the knowledge score, employment, sex, age and education among particleboard workers in Ethiopia

Variable	Univariate analysis		Multivariate analysis	
	β	*p* value	β	*p* value
Intercept			−1.5	0.07
Employment (0 = Temporary 1 = Permanent)	2.4	*<* 0.001	1.7	0.011*
Sex (0 = Male 1 = Female)	−0.98	0.203		
Age (0 = 19–27 1 = 28–50)	0.4	0.307		
Education (0 = grade 1–10, 1 = Vocational and above)	2.1	*<* 0.001	0.96	0.112

Further analysis using multivariate regression showed that employment is significantly associated with the knowledge score while adjusting for education. When age and sex were included in the multivariate analysis, the results were the same.

### Attitudes related to chemical hazards

Higher proportion of permanent workers had significantly positive response than temporary workers on 6 of 11 topics on attitude related to reduction of chemical hazards and the general working environment (Table 4). A higher proportion of temporary (82%) than permanent workers (38%) believed that all PPE has the same level of protection. For four attitude-related questions there were no significant difference between permanent and temporary workers.

**Table 4:** Attitudes of particleboard workers about overall workplace hazards and safety in Ethiopia

Variable	Total *n* (%)	Employment status (*n* = 159)		*p* value
		Permanent (*n* = 121) *n* (%)	Temporary (*n* = 38) *n* (%)	
Workplace is hazardous to health	132(83)	100(83)	32(84)	1
I should use PPE during work	155(98)	120(99)	35(92)	0.06
Employer has responsibility to reduce exposure of hazards	143(90)	109(90)	34(90)	1
All PPE has same level of protection	77(48)	46(38)	31(82)	*<* 0.001
I should follow workplace safety rule	143(90)	118(98)	25(66)	*<* 0.001
PPE is relevant in workplace	153(96)	119(98)	34(90)	0.04
Employer should supply PPE	153(96)	118(98)	35(92)	0.296
I should always use PPE	148(93)	112(93)	36(95)	0.925
Safety training is relevant	137(86)	113(93)	24(63)	*<* 0.001
Safety professionals are relevant	150(94)	118(98)	32(84)	0.007
Feel satisfied with my work	117(74)	96(79)	21(55)	0.006

### Practices of workers related to chemical hazards

Provision of PPE, as perceived by the permanent workers varied from monthly to annually and many workers did not know about the schedule of PPE distribution (Table 5).

**Table 5:** Schedule for provision of personal protective equipment as reported by the permanent particleboard workers (*n* = 121)

Type of PPE	Frequency of distribution(*n* = 121)				
	I do not know	Annually	Semi-annually	Quarterly	Monthly
Safety glass	37	22	30	30	2
Face mask	32	22	30	35	2
Gloves	58	20	25	16	2
Coverall	11	26	79	5	–

From the total 159 workers 103 (66%) were using at least one type of PPE during work. All permanent workers responded that the factory provides PPE and 98 (81%) workers reported they used at least one PPE during work irrespective of its quality. Among temporary workers, only 3 (7.9%) reported that the factory management provides PPE, while the remaining 35 (92.1%) did not get PPE from the factory. They reported using other options like buying from the market. Seven (18.4%) temporary workers reported using at least one type of PPE during work irrespective of its quality. Neither permanent nor temporary workers were using the full set of PPE during work. The practice of PPE use during work among permanent workers was significantly higher than among temporary workers (81% vs 18.4%) (*p <* 0.001).

To use PPE, permanent workers were motivated by supervisor 58 (54%), by safety personnel 12 (11%), by colleagues 8 (7.4%), self-motivation 76 (70%) and health professionals 2 (1.8%). The reasons for not using any type of PPE were reported to be lack of access (59%), lack of knowledge of its importance (33%), not comfortable (3.9%), not useful (1.9%), and 1.9% said that PPE was easily damaged.

During the workplace visit we observed that the PPE used did not have any specification like production date, intended use and protection level. This makes it difficult to evaluate the quality of the PPE. There was also a common understanding among the workers that the available face mask has no protective value.

The visiting health institution for medical check-up was reported by 25% of the permanent workers and 37% of the temporary workers, which was not statistically different. Attending some safety training about occupational hazards was reported by 10 and 0% of the permanent and temporary workers respectively. However, both permanent and temporary workers reported that there was no scheduled or regular training about occupational hazards in the factories.

### Information from administrative personnel

The information collected from 13 administrative personnel was obtained from persons with different positions (general manager, deputy manager, production manager, technique manager, quality control, safety coordinator, logistic and supply and health professional). All stated dust and formaldehyde as chemical hazards found in the factory. They also mentioned that the availability of safety guidelines, good lighting, good ventilation and good communication between workers and the employer reduces exposure to chemical hazards. Seven responded that job rotation reduces exposure to chemical hazards. All said that PPE is given to every worker, but the schedule they reported varied, also within the factories. Six respondents thought that all PPE has the same level of protection, and that the factory simply purchases PPE that is available in the market without any quality consideration. Six respondents stated that new PPE is given immediately to the worker when they lose or damage it. The administrative personnel stated that there is regular supervision to obtain safe working practices in the factory. They also mentioned that the safety committee assures the supply of PPE and creates awareness among workers. Ten individuals stated that safety training is given to workers. However, the response on the frequency of the training varies.

## Discussion

Permanent workers have more knowledge about chemical and other occupational hazards than temporary workers in particleboard factories. Of the total workers, 82% know some type of chemical hazards. The permanent workers were more interested in controlling exposures from hazardous chemicals than the temporary workers. Almost all permanent workers and few temporary workers used at least one PPE during work. However, the quality of the PPE was questionable and only few temporary workers reported that PPE was provided by the factory.

In this study, permanent workers had more knowledge about chemicals and other hazards than temporary workers. This might be because temporary workers start their jobs as helpers i.e. assisting permanently hired workers without prior training on occupational health and safety. For example, helpers in the chemical section assist the chemist in handling bags, cleaning the machines and controlling filters, pumps, hoses and blenders. They also check the glue kitchen and report when there is anything out of control. Our finding is in line with a descriptive study done in Nigeria among 200 textile workers which shows that permanent employment was a determinant for knowledge about workplace hazards [44]. Several studies on the association between injuries and employment status have shown that the risk of injuries among temporary workers is higher compared to that among permanent workers [38–42]. High risk of injuries among temporary workers might indicate the workers have less knowledge about different occupational hazards and the employer has given them less attention. In our study 82% of the total workers knew some types of chemical hazard. This is in line with a study done in Nigeria among 200 dye workers, which indicated that 74% had knowledge about workplace hazards [44]. This Nigerian study also indicated that permanent employment was a determinant for knowledge of workplace hazards when adjusted for education [44], mirroring a finding in our present study.

High educational status was associated with a high knowledge score. This finding is also in line with a cross-sectional study done in Nigeria on 290 health care workers showing that the level of education is related to knowledge about workplace hazards [36]. A study done in Colombia also supports these finding as it indicates that level of education was a determinant for knowledge of dengue disease and its transmission [29].

The temporary workers did not show the same attitude to reducing chemical hazards in the factory as the permanent workers. The finding is in line with a study in Nigeria showing that permanent employment was a determinant for attitude of workers towards workplace hazards [44]. In our study, the majority of the workers’ attitudes about the means and how to behave to reduce chemical hazards was high (74% and above) and this finding is also in agreement with a study done in Nigeria, which indicates that 81% had a positive attitude about the workplace hazards and their control measures [44].

In our study 66% of the workers used at least one type of PPE during work. However, to protect from the workplace hazards, the workers need to wear a complete set of PPE. There was a common understanding among the workers that the available PPE, mainly face mask, did not have any protective value. On top of this, temporary workers were not getting a PPE supply. This perception of lacking supply of PPE probably has its own negative effect on the practice of using PPE during work. These findings are in line with a study done in India and Ethiopia showing that non-use/use of safety material was due to unavailability/availability [28, 30]. The quality of the PPE was another bottleneck problem for utilization. The respondents who have access to that PPE reported that the PPE was easily damaged and out of use within a short period of time. Due to this they don’t believe it protects from exposure. This perception is similar to the perception of the workers in Nigeria, which indicates that workers think the PPE is useless in terms of hazard protection [33]. Our finding is also in agreement with different studies demonstrating workers’ lower practice of PPE due to low access and unsuitability in different work settings [31, 32, 34]. However, a study done in Ethiopia among textile workers showed better frequency of PPE use, which is contrary to our finding. The reasons mentioned for better frequency in that study were: difference in workplace conditions, different level of awareness, difference in data collection tool and availability of PPE [28].

Permanent workers have better practice than temporary workers. This finding is in line with the results from a cross-sectional study among 560 Ethiopian textile workers regarding knowledge and safety [35]. In our study only 10% of the permanent workers and none of the temporary workers attended safety training, which might affect PPE use [27, 35]. Although many of the workers (87%) know some chemical hazards, their practice was poor due to the negative attitude about the existing PPE in terms of hazard protection. This finding is in line with a cross-sectional study in India among 216 garment workers indicating a wide gap between their knowledge and practice of use of PPE during work [31].

Permanent workers’ response on the schedule of different PPE was inconsistent and differed from the responses obtained from administrative personnel in the same factories. Some of the respondents even did not know the schedule of PPE supply. This might indicate irregularities in the supply of PPE. On top of this, PPE such as face masks were not marked with quality information and with such lacking information it was difficult to evaluate its actual quality. In our study, 56% of the workers were vocationally trained which is different from other studies, where the educational status of the workers was either primary [28] or secondary [35].

Information collected from the administrative personnel indicated that they were aware of the existence of different hazards like dust and formaldehyde. However, there were no safety personnel that could monitor and assure safety practice in the factories. This has an impact on the technical requirements to consider when ordering PPE. The administrative workers in charge of supplying logistics and equipment to the factory workers also purchase safety materials, however without the competence needed to order PPE according to the required quality. Most of the administrative personnel believed that all PPE has the same level of protection.

Findings of this study can inform the employers to give equal attention both for permanent and temporary workers’ safety and health protection. Employers may undertake such strategies as eliminating or minimizing chemical exposures in the physical work environment through engineering controls or redesigning production processes. Furthermore, providing safety and health training (both pre-employment and periodic) and instituting other necessary administrative controls (e.g. job rotation, facilities for meals and rest breaks) could help in reducing chemical exposures. Although the last resort in the hierarchy of controls, provision of adequate PPE is necessary to protect workers. The study findings might also help policy makers to expand the KAP knowledge and promote the safety and health of workers in the wood industry. For future research, an exposure assessment intervention study could be considered.

A strength of the study is the high response rate. The limitations of the cross-sectional study design will presumably not affect the reliability of the data collected because the information has no health variable and does not study any causal relationship between exposure and health. However, it could clearly have been an advantage to obtain information more than only once. The questionnaire was developed by reviewing KAP articles [26, 35, 43] and a pre-test was done before data collection. This may increase the validity of the study. Qualitative information and self-reports collected from both production workers and management personnel might expand the KAP, both from the worker and management perspective. However, the workplace assessment could have been improved by systematically collected objective data on for instance the use of PPE. This is an option for future studies. Although the data collection was performed one by one in a place without others listening, there might be still a response bias. Study participants can either disclose or hide the information. This study was targeted on large wood manufacturing industries. It might be difficult to generalize the results for small scale, medium scale and less formal wood manufacturing industry, for which the situation could be different.

## Conclusion

This study shows that most workers know about chemical hazards, associated health effects, and preventive measure to reduce chemical exposures. Permanent workers reported more safety-conscious responses to attitude-related questions. Use of PPE was higher among permanent workers; however, temporary workers were not always provided with PPE. Both permanent and temporary workers should be equally privileged in all the safety and health services delivered by the workplace. A systematic qualitative study is needed for future work. This could be combined with an exposure assessment intervention study.

## Additional file

Additional file 1: English-language data collection tool. The full data collection questionnaire for this paper is added as a supplementary file. (DOC 120 kb)

## Abbreviations

CI: Confidence Interval
IRB: Institutional Review Board
KAP: Knowledge Attitude and Practice
M/F: Male/Female
N/Y: No/Yes
PPE: Personal Protective Equipment
*SD*: Standard deviation
US: United States
