# Supplementary material for: Knowledge, attitude and practice related to chemical hazards and personal protective equipment among particleboard workers in Ethiopia: a cross-sectional study
Source: BMC Public Health. 2019 Apr 27;19:440. doi: 10.1186/s12889-019-6807-0 (PMC6487067; doi:10.1186/s12889-019-6807-0)
Supplement: Supplementary file 1 — English-language data collection tool. The full data collection questionnaire for this paper is added as a supplementary file. (DOC 120 kb) [file 12889_2019_6807_MOESM1_ESM.doc]

English Version 1 questionnaire for the Assessment of Knowledge, attitude and practice related to chemical hazards and personal protective equipment among particleboard workers in Ethiopia.

| S.No. | Category | Questions | Response |
| --- | --- | --- | --- |
| 1 | Sociodemographic | 1. Code |  |
|  |  | 1. Site | 1. Maichew B. Hawassa |
|  |  | 1. Sex | 1. Male B. Female |
|  |  | 1. Age (Years) |  |
|  |  | 1. Highest grade completed? |  |
|  |  | 1. What is your Profession? |  |
|  |  | 1. Type of employment? | 1. Permanent 2. Temporary |
|  |  | 1. Working section? | A. Chipping B. Flaking  C. Chemical D. Forming  E. Trimming F. Sanding G. Others, |
|  |  | 1. Service year in this factory? |  |
|  |  | 1. Service year in another factory (if applicable) |  |
|  |  | 1. Total working hours per day |  |
| 2 | Knowledge | 1. Do you know the type of chemical hazards arsing from this factory? | 1. Yes 2. No, Skip to Q# 14 |
|  |  | 1. If yes for Q.# 12, can you list some of them? | 1. Dusts   B. Formaldehyde  C. Other, specify--------- |
|  |  | 1. Do you know a health hazards that could arise from chemical exposures? | 1. Yes 2. No, Skip to Q# 16 |
|  |  | 1. If yes, for Q.# 14 can you mention some of the health problems? |  |
|  |  | 1. Do you know hazards other than chemicals? | 1. Yes 2. No |
|  |  | 1. Is emergency exit important | 1. Yes 2. B. No |
|  |  | 1. Is break time during work important to reduce exposure to chemical hazards? | 1. Yes 2. No |
|  |  | 1. Is job rotation important for reducing exposure to chemical hazards? | 1. Yes 2. No |
|  |  | 1. Do you know about material safety data sheet? | 1. Yes 2. No, Skip to Q# 22 |
|  |  | 1. If yes, for Q.# 20, Can you explain it? |  |
|  |  | 1. Do you understand the sign and symbols posted in the wall? | 1. Yes 2. No, Skip to Q# 24 3. Not applicable |
|  |  | 1. If yes, for Q.# 22, can you explain the symbols with their meaning? |  |
|  |  | 1. Do you know any work place safety rule? | 1. Yes, describe some 2. No |
|  |  | 1. Do you know what personal protective equipment is? | 1. Yes 2. No, Skip to Q#29 |
|  |  | 1. If yes, for Q.# 25, can you mention those you know with their use? |  |
|  |  | 1. Is personal protective equipment releavant in your work place? | 1. Yes 2. No, Skip to Q# 29 |
|  |  | 1. If yes, for Q.# 25 who should wear personal protective eqipment? |  |
|  |  | 1. Do the factory/employer has an obligation to maintain workers safety and health? | 1. Yes 2. No 3. I do not know |
|  |  | 1. Do you have any source of information about occupational health and safety at work place? | 1. Yes 2. No, Skip to  Q# 32 |
|  |  | 1. If yes for Q.# 30 what are the sources of informations you know? |  |
| 3 | Attitude | 1. Do you believe that working in this factory is hazardous? | 1. Yes 2. No |
|  |  | 1. Do you think as an employee you should always use PPE during work? | 1. Yes 2. No |
|  |  | 1. Do you believe that your employer is responsible for reducing exposure to chemical hazards? | 1. Yes 2. No |
|  |  | 1. Do you think that all PPE has the same level of protection? | 1. Yes 2. No |
|  |  | 1. Do you believe that you should follow safety rules? | 1. Yes 2. No |
|  |  | 1. Do you believe that personal protective equipment relevant in work? | 1. Yes, justification. 2. No |
|  |  | 1. Do you believe that employer should supply PPE? | 1. Yes 2. No |
|  |  | 1. Do you believe that safety training is relevant for workers? | 1. Yes 2. No |
|  |  | 1. Do you believe that Health/safety professional are relevant in your work place? | 1. Yes 2. No |
|  |  | 1. Do you feel satisfied with your work? | 1. Yes 2. No |
| 4 | Practice | 1. Where do you get personal protective equipment? |  |
|  |  | 1. Do you use personal protective equipment during work? | 1. Yes 2. No, Skip to Q#49 |
|  |  | 1. If yes, for Q. # 43, why you use them? | A. They protect from hazard  B. The factory ordered us to use them  C. I am not sure  D. Others, specify |
|  |  | 1. If no, for Q. # 43, why do not you use them? |  |
|  |  | 1. Who encourages you to use personal protective equipment? | A. Supervisor  B. Safety personnel  C. Collegues  D. Others, specify |
|  |  | 1. How often is personal protective equipment supplied?(Specify by type) |  |
|  |  | 1. What do you do if your personal protective equipment is lost/torn? | A.Buy new one  B. Request new one  C. Nothing to do  D. Others, specify |
|  |  | 1. Do usually made medical check ups? | A.Yes  B.No |
|  |  | 1. Do you usually attend safety training? | A.Yes  B.No |
| Thank you for your cooperation !!!! | | | |

English Version 2 questionnaire for the Assessment of Knowledge, attitude and practice related to chemical hazards and personal protective equipment of particleboard workers in Ethiopia from administrative personnel perspectives.

| S.No. | Questions | Response |
| --- | --- | --- |
| 1 | Code |  |
| 2 | Site | A. Maichew B. Hawassa |
| 3 | Age |  |
| 4 | Sex | A. Male B. Female |
| 5 | Responsibility |  |
| 6 | Educational level |  |
| 7 | Profession |  |
| 8 | What are the chemical hazards occurred in your factory? |  |
| 9 | What do you do when workers face hazards from the work environment? |  |
| 10 | Is there safety committee in your factory? | A. Yes  B. No, Skip to Q#12 |
| 11 | If yes, for Q. # 10 what is their function? |  |
| 12 | Can availability of safety guideines redues exposure to chemical hazards? | A.Yes B. No |
| 13 | Can job rotation reduce exposure to chemical hazards? | A. Yes B. No |
| 14 | Can break time during work reduce exposure to chemical hazards? | A.Yes B. No |
| 15 | Is good communication important in reducing exposure to chemical hazards at work? | A.Yes B.No  C. I do not know |
| 16 | Is proper lighting important in reducing exposure to chemical hazards at work? | A.Yes B.No  C. I do not know |
| 17 | Is good ventilation important in reducing exposure to chemical hazards at work? | A.Yes B.No  C. I do not know |
| 18 | Do you supply safety material to workers? | A.Yes, which type?  B. No, why? |
| 19 | How often do you supply personal protective equipment? |  |
| 20 | Do you think all type PPE have the same level of protection? | A.Yes B.No |
| 21 | What are the consideration when you purchase PPE? |  |
| 22 | What do you do if workers lost/torn their personal protective equipment? |  |
| 23 | Is there any safety training given for workers? | A.Yes  B.No, Skip to Q#25 |
| 24 | Id yes for Q# 23,  How often and who gives the training? |  |
| 25 | Is there regular supervision and follow up to workers on safety rules and practices? | A.Yes B.No |
| Thank you for your participation! | | |
